# Acalculous Cholecystitis in a Seven-Year-Old Girl With Epstein-Barr Virus Infection

**DOI:** 10.7759/cureus.19774

**Published:** 2021-11-20

**Authors:** Jeffrey Rein, Brent Watkins

**Affiliations:** 1 Pediatrics, Southern Arizona Veterans Affairs (VA) Health Care System, Tucson, USA; 2 Pediatrics, The University of Arizona, Banner-Diamond Children's Medical Center, Tucson, USA; 3 Pediatrics, Tanque Verde Pediatrics, Tucson, USA

**Keywords:** sterile pyuria, mononucleosis, acalculous cholecystitis, pediatrics patient, epstein-barr virus

## Abstract

Epstein-Barr virus (EBV) infection with associated acute acalculous cholecystitis (AAC) has been reported in 18 pediatric patients. Our case is that of a seven-year-old girl with acute EBV infection and associated AAC.

## Introduction

Infectious mononucleosis is a common illness in the pediatric population and Epstein-Barr virus (EBV) is the most common etiologic factor. It can have a wide range of manifestations, from asymptomatic to systemic, severe disease. Since first being reported in 2007 [1], there have been reports of 17 additional pediatric patients with acute acalculous cholecystitis (AAC) as a result of EBV infection [2-16]. We present an additional case in which AAC was the clinically dominant manifestation of acute EBV mononucleosis. Differential diagnosis and workup of pediatric AAC are discussed, as well as evidence from our case and the literature in support of a non-invasive, expectant management approach.

## Case presentation

A previously healthy seven-year-old girl was admitted with one week of tactile fevers, right upper quadrant pain and vomiting. In the week prior to admission, she was seen twice where laboratory testing demonstrated a white blood cell count of 24,000, pyuria and microhematuria. She was prescribed first trimethoprim/sulfamethoxazole then cephalexin for presumed urinary tract infection, then sought further care in the US due to persistent symptoms.

On presentation at our hospital, the patient complained of right upper quadrant and periumbilical pain. She appeared well. Physical examination was significant for shotty cervical lymphadenopathy and abdominal tenderness in the right upper quadrant and periumbilical regions, and the liver was palpable 2 cm below the costal margin. External genitalia were unremarkable.

Laboratory evaluation revealed leukocytosis of 41,200/μl (90% lymphocytes), elevated transaminases (ALT 114 IU/L) and hyperbilirubinemia (total 1.8 mg/dL, direct 1.1). LDH was elevated to 732 U/L with normal uric acid. Peripheral blood smear showed reactive lymphocytes and smudge cells, without blasts. Urinalysis demonstrated sterile pyuria and microhematuria. Blood cultures were sterile.

**Figure 1:**
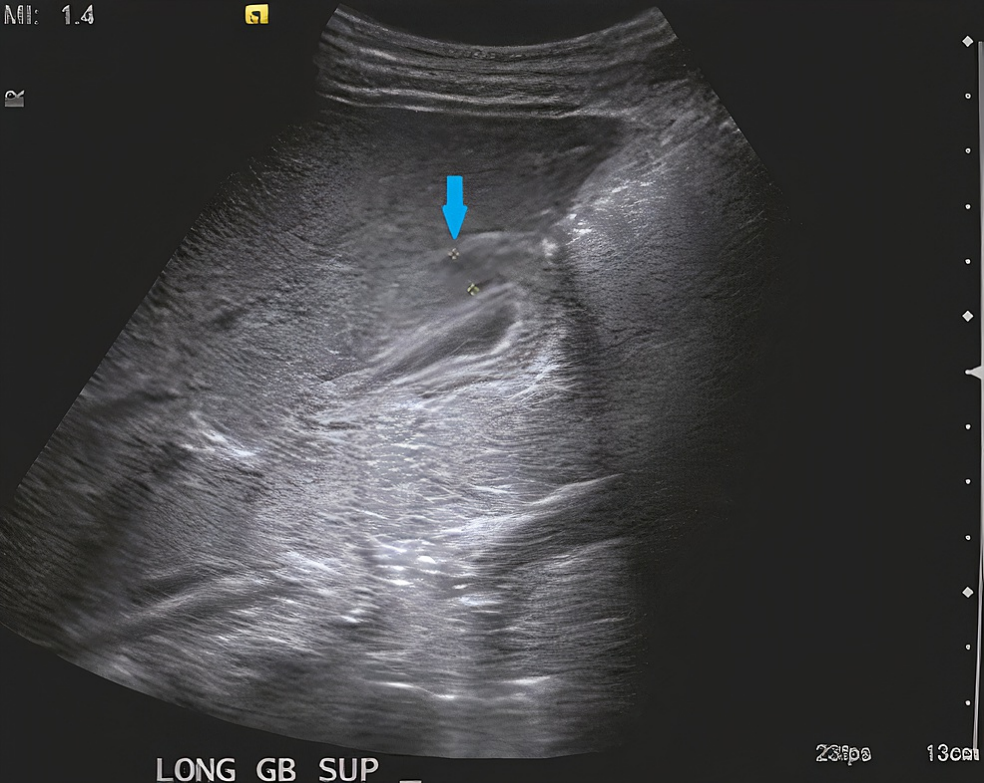
Abdominal ultrasound demonstrating gallbladder wall (blue arrow) of thickness 0.73 cm (yellow dashed crosses).

Abdominal ultrasound showed pericholecystic fluid and gallbladder wall thickening without dilated biliary ducts or gallstones, consistent with AAC (0.73 cm gallbladder wall thickness; Figure 1). The ultrasound also demonstrated splenomegaly (10.3 cm). Hepatic echotexture and architecture were normal as were the kidneys, bladder and collecting system.

Heterophile antibodies were negative. IgM and IgG against the Epstein-Barr viral capsid antigen were positive, as was IgG against early D antigen. Nuclear antigen IgG was negative. Serologic assays for infection with hepatitis A, B and C were negative.

The patient’s symptoms improved and she was discharged on hospital day 4, with falling transaminases and leukocyte count. At the outpatient clinic one week after presentation, the patient was asymptomatic, with a normal exam. Laboratory testing demonstrated a white blood cell count of 16,000 with lymphocyte predominance, and an ALT of 80. Bilirubin and urinalysis were normal. The family declined repeat sonography and was subsequently lost to follow-up.

## Discussion

Our patient had many common manifestations of EBV mononucleosis, including lymphocytosis, splenomegaly, adenopathy and hepatitis. Clinically occult genitourinary involvement, previously reported in EBV, may have accounted for our patient’s transient sterile pyuria and hematuria [17].

Acute acalculous cholecystitis in a previously well child may be attributable to infectious agents such as *Coxiella burnetii* (Q fever) and *Leptospira* species (leptospirosis). Both of these agents may produce liver and urinalysis abnormalities as well. Other infectious agents have been implicated in hepatobiliary pathology including AAC. In immunocompromised hosts (with concurrent HIV infection, for example) cytomegalovirus and Mycobacterium avium are important considerations although typically accompanied by clinical and radiographic signs of biliary obstruction, which our patient lacked.

In the absence of historical antecedents of immunocompromise or contact with animal vectors for *C. burnetii* or Leptospira, EBV was considered. Testing for heterophile antibodies was negative. IgM and IgG against the Epstein-Barr viral capsid antigen were positive, as was IgG against early D antigen. Nuclear antigen IgG was negative. These results are diagnostic of acute EBV infection, in our patient’s case complicated by acute acalculous cholecystitis.

Nearly every organ system has been documented to be affected in EBV mononucleosis. Clinical evidence of AAC, however, is not common. There have been 18 reported cases of pediatric patients with symptomatic EBV-associated AAC [1-16] although radiographic stigmata may be found in up to 25% of cases of pediatric acute EBV [18]. Surgical and antibacterial therapy have been proposed [6] but no reports of adverse outcome have been associated with expectant management alone. Our case demonstrates the value of considering EBV in the differential diagnosis of pediatric acalculous cholecystitis, with subsequent avoidance of unnecessary and potentially harmful interventions such as cholecystectomy or antibacterial therapy.

## Conclusions

Acute EBV infection may cause symptomatic acalculous cholecystitis in children. Despite the limited number of case reports in the literature, radiographic surveys of children with acute EBV suggest that this condition may be more common than generally believed. Increased awareness of this atypical manifestation (AAC) of a common disorder (acute EBV), with appropriate testing to establish the etiologic diagnosis, may permit providers to avoid needless and potentially harmful interventions. 
